# Stoichiometry deviation in amorphous zirconium dioxide

**DOI:** 10.1039/c9ra01865d

**Published:** 2019-05-24

**Authors:** Michael J. D. Rushton, Iuliia Ipatova, Lee J. Evitts, William E. Lee, Simon C. Middleburgh

**Affiliations:** Nuclear Futures: Materials, Bangor University Bangor LL57 1UT UK s.middleburgh@bangor.ac.uk; Department of Materials, Imperial College London London SW7 2AB UK

## Abstract

Amorphous zirconia (a-ZrO_2_) has been simulated using a synergistic combination of state-of-the-art methods: employing reverse Monte-Carlo, molecular dynamics and density functional theory together. This combination has enabled the complex chemistry of the amorphous system to be efficiently investigated. Notably, the a-ZrO_2_ system was observed to accommodate excess oxygen readily – through the formation of neutral peroxide (O_2_^2−^) defects – a result that has implications not only in the a-ZrO_2_ system, but also in other systems employing network formers, intermediates and modifiers. The structure of the a-ZrO_2_ system was also determined to have edge-sharing characteristics similar to structures reported in the amorphous TeO_2_ system and other chalcogenide-containing glasses.

## Abstract 2

The accommodation mechanism for excess oxygen in amorphous ZrO_2_ is identified using state-of-the-art methods: employing reverse Monte-Carlo, molecular dynamics and density functional theory together. Excess oxygen is predicted to enter amorphous ZrO_2_ exothermically from O_2_.

## Graphical abstract

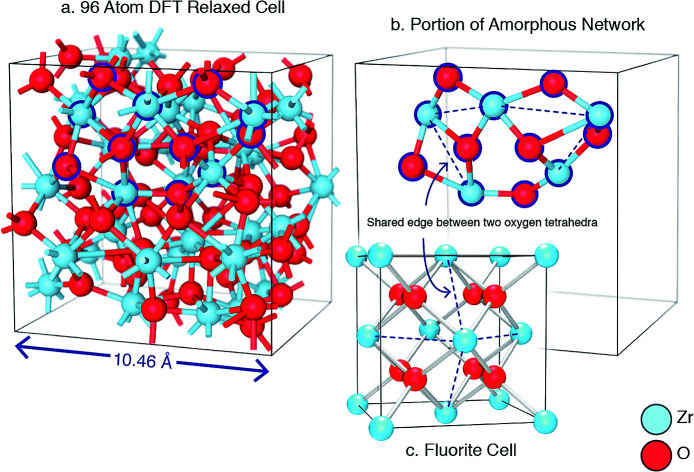

## 1 Introduction

Zirconium dioxide (ZrO_2_) is a widely used and important material in engineering applications including thermal barrier coatings within the aerospace industry^1^ and transistor components in electronic engineering.^2^ In the nuclear energy sector ZrO_2_ has been used as an inert matrix nuclear fuel material^3^ and has been investigated as a potential nuclear waste-form.^4^ Additionally, ZrO_2_ is the protective layer formed when zirconium-based alloys are exposed to air and steam (as in a nuclear reactor environment^5,6^). The oxide has three major polymorphs:^7^ the low temperature (*T* ≲ 1440 K) monoclinic; intermediate temperature (1440 ≲ *T* ≲ 2640 K) tetragonal; and high temperature (*T* ≲ 2640 K) cubic. A metastable amorphous phase has also been widely reported^8–12^ and studied for potential use in electronics as resistive random-access memory (RRAM) devices^13^ and as a replacement for SiO_2_ in complementary metal-oxide-silicon (CMOS) devices.^14,15^

The radiation damage and potential amorphization of zirconia is of particular importance when considering the behaviour of zirconium alloys in corrosive, nuclear environments – such as those found in a typical light water reactor (LWR). ZrO_2_ is the passivating layer on such alloys and is extremely tolerant to radiation damage,^16^ remaining crystalline (in its cubic stabilized state) at low temperatures and high fluence^17^ – only amorphizing when the grain size is extremely small (∼50 nm).^18^ What has not been fully understood is the potential formation of amorphous films or phases at grain boundaries^19^ when exposed to radiation or corrosive environments, and the effect these may have on the behaviour of the bulk ceramic in terms of mechanisms that limit the protective nature of the oxide.

The formal charge of the Zr ion in ZrO_2_ is not readily increased from 4+ to 5+ as to do so would require electron removal from a new orbital (an ionization energy of ∼46 eV). In other similar cation systems such as MgO,^20^ BeO,^21^ (Ba/Sr)ZrO_3_,^22^ and CeO_2_,^23^ where the cation is at its highest formal charge, it has been predicted that the peroxide ion (O_2_^2−^) can accommodate an increase in oxygen content without the need to oxidize a cation in the system. Importantly, the formation of O_2_^2−^ ion has been experimentally observed through Raman spectroscopy in both BaZrO_3_ and SrZrO_3_ as a route to accommodate hyper-stoichiometry.^22^ Hypo-stoichiometric ZrO_2−*x*_ has been widely studied and it has been observed that non-monoclinic phases can be stabilized by the resulting defects and non-stoichiometry.^24^ Electronic defects may also be a route for charge compensating deviations in oxygen stoichiometry.^25,26^

The periodic nature of crystals means that any interruption of their structures is easily described in terms of defects. For instance point-defects such as vacancies and interstitial atoms can be unambiguously defined in terms of their species, effective charge and crystal site, and communicated compactly using Kröger–Vink notation.^27^ Crystals contain only a limited number of symmetrically distinct atomic sites and are described in terms of a lattice and a generally small repeating atomic motif. These symmetry relationships greatly reduce the number of atomic environments that must be considered to understand the defect chemistry of a crystalline material.

Later in this paper we describe how the structure of amorphous ZrO_2_ accommodates changes in the oxygen stoichiometry. In the crystalline material these changes would be easily described in terms of point defects. However, the lack of long-range order that characterises the amorphous state, means that traditional definitions of point-defects break down when there is no lattice to act as a frame of reference. However, certain topological features in the amorphous state often have similarities with point-defects^28^ and we will draw such comparisons where appropriate.

In this work, the potential for deviations in stoichiometry in amorphous ZrO_2_ is investigated using a combination of molecular dynamics and reverse Monte-Carlo calculations using classical potentials and density functional theory (DFT). Both classical and quantum mechanical methods have been used previously to study amorphous systems: effective potential methods having the benefit of being able to simulate large, complex supercells with tens of thousands of atoms,^29–31^ while the greater physical rigour of DFT methods and its quantum mechanical description of electronic properties additionally allows subtle chemical effects due to charge transfer and electronic defects to be considered.^32,33^

## 2 Methodology

For this work a number of methodologies have been combined to generate representative amorphous structures and then extract relevant property and structural information.

The following section outlines the general workflow used:

(1) Melt-quench: Classical molecular dynamics simulations were used to represent the process of rapidly cooling a system from the molten state to induce an amorphous state. This first step was conducted for a relatively large simulation box which contained 96 000 atoms (in the stoichiometric case). More details of this step are given in Section 2.1.

(2) Reverse Monte-Carlo: In this step much smaller simulation cells (96 atoms when stoichiometric) were generated using the Reverse Monte-Carlo (RMC) method from the description of the amorphous state predicted in the previous step. A particular aim of this study was to compare the electronic structure of crystalline ZrO_2_ with that of the amorphous state. This required the use of the DFT method (see the next step). The computational expense of DFT means that, due to their large size, the amorphous structures from step 1 cannot be used directly in DFT calculations. Instead, information obtained from the MD simulations such as density, pair-correlation functions and bond angle distributions, are extracted and used to parametrise RMC simulations. In these, the small systems are refined, through a series of stochastic atom moves, to produce structures that are consistent with the structural information extracted from step 1. A detailed description of this appears in Section 2.2.

(3) DFT: Finally the small cells were structurally relaxed using DFT energy minimisation. This allowed the structure and properties of the system to be better understood and compared with the various polymorphs of crystalline ZrO_2_. The DFT method employed is given in Section 2.3.

The method described here has some similarities with previous work by Vanderbilt *et al.*,^34^ Zhao^14^ and Ceresoli.^35^ They used a melt-quench technique to study amorphous ZrO_2_ and claimed good agreement with experimentally obtained values for the dielectric constant of the material. The melt-quench used by Vanderbilt *et al.* was performed on small simulation cells (96 atoms) using DFT MD. Here we use much larger simulation cells (96 000 atoms) and classical potentials to perform melt-quenches at much slower quench rates (4 × 10^11^ K ps^−1^*versus* 3.4 × 10^14^ K s^−1^ of the previous work).

The details of each of the MD, RMC and DFT steps will now be described.

### 2.1 Molecular dynamics – melt quench

The following procedure was used to simulate rapid cooling from the molten state in order to mimic the type of experimental melt-quench often used to produce amorphous materials. The starting structure for all quenches was based on a 20 × 20 × 20 super-cell of cubic zirconia. As will be described in Section 2.2, hypo- and hyper-stoichiometry was introduced by adding or removing oxygen atoms to this supercell as necessary. The same quench procedure was used for all stoichiometries which is now described. All MD calculations were performed using the 11 Aug 2017 version of the LAMMPS code^36^ and an integration time-step of 1 fs was used throughout.

The cubic form of ZrO_2_ is associated with high temperatures, consequently, the simulation cell was initially equilibrated at a temperature of 2700 K for 100 ps. In order to quickly stabilise the system's temperature and reach the density predicted by the chosen potential model (described below), a Berendsen thermostat and barostat were used (with relaxation times of 0.1 ps and 1 ps respectively).^37^ This was followed by another 50 ps of MD at 2700 K which now used the Nosé–Hoover thermostat and barostat^38–40^ (to allow more representative sampling of the NPT thermodynamic ensemble). The Nosé–Hoover thermostat had a relaxation time of 0.01 ps and the barostat used 0.1 ps. These were maintained for the remainder of the quench. An MD barostat controls pressure by adjusting cell parameters to achieve a target pressure, here this was 0 GPa and, in-line with the isotropic nature of amorphous materials, the barostat was set to maintain a cubic geometry throughout.

To remove any bias due to the crystalline starting structure, the system was heated to a high temperature of 5000 K. This was achieved by ramping the temperature during a 50 ps MD run. Once at temperature, another 50 ps of MD was performed, this can be thought of as the ‘melt’ section of the melt-quench, even though it takes place at a much higher temperature than the experimentally measured melting point of zirconia (2988 K (ref. 41)). Before commencing the quench, the radial distribution function of system was checked to ensure a molten state had been achieved.

**Fig. 1:**
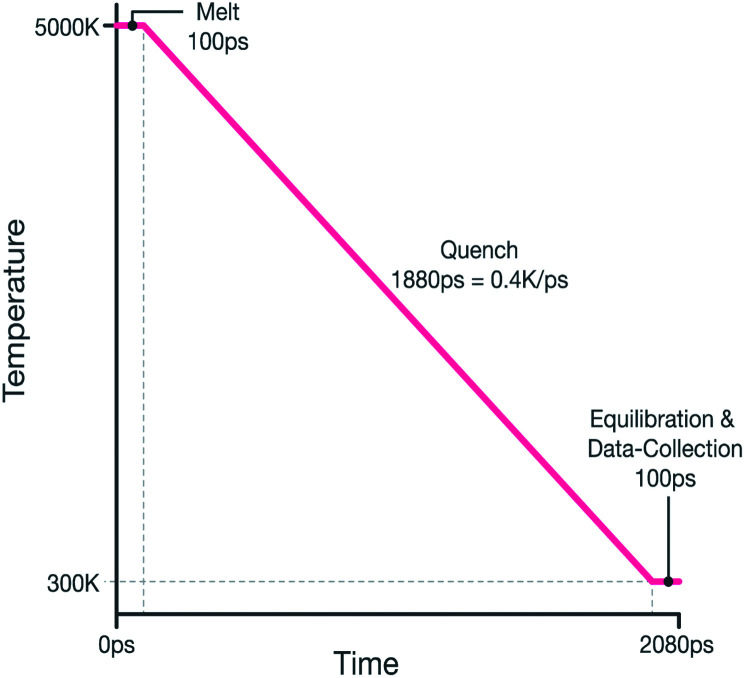
Graphical representation melt-quench procedure.

The system was then quenched from 5000 K to 300 K by reducing the thermostat's temperature linearly during a 1.88 ns NPT MD run, therefore giving an effective quench rate of 0.4 K ps^−1^. The melt-quench procedure is summarized graphically in Fig. 1.

On reaching 300 K, the data to be used as inputs to the RMC method were collected. This was achieved by performing a final 100 ps MD run during which pair-correlation functions for the Zr–O, Zr–Zr and O–O species pairs were calculated and time averaged over the final 50 ps of the run by collecting data at 5 ps intervals. Atomic densities and relevant bond-angle distributions were also averaged in this way.

The many body potential model derived by Liu, Cooper *et al.* was used to describe interatomic forces during all MD runs.^42^ Although this potential provides an excellent high temperature description of the tetragonal and cubic forms of ZrO_2_ and the transition between them, it must be recognized that the potential model does not reproduce the monoclinic phase.

### 2.2 Reverse Monte-Carlo

Reverse Monte-Carlo is a structural refinement technique which aims to generate model atomic systems, which are consistent with a set of constraints and structural data. RMC was originally developed to help interpret experimental diffraction data obtained from amorphous systems.^43–45^ Of relevance here, Winterer used RMC to examine the structure of amorphous and monoclinic ZrO_2_ using experimental data obtained using the EXAFS technique.^46^

Diffraction experiments for crystals can be analysed to give a unit-cell and consequently provide an unambiguous description of the structure. By comparison, the shape-factor and radial distribution functions obtained from the diffraction of amorphous materials provide averaged data about the pair separations of atoms in the material. This means that it is not possible to define a single atomic structure from amorphous diffraction data as multiple structures may be consistent with a particular set of radial distribution functions (RDFs). RMC provides a method to generate atomic configurations that aim to match experimental RDFs. Furthermore, by including additional physical constraints on the material structure such as bond lengths, bond angles and coordination number limits, configurations may be generated that are more physically rigorous than fitting to RDFs alone. A full description of the method is provided elsewhere however a simple description of its operation is now given.^47,48^

A set of atoms with the desired composition and density are assigned random positions in a simulation box. The RDF for this model system is calculated and compared with the experimental RDF. The root mean squared (RMS) difference between the two shows the goodness of fit between model and experiment. An atom in the model is then randomly moved and the comparison is made again. If the fit is improved (indicated by a decrease in the RMS difference), the move is accepted – if not, the move may still be accepted based on the Metropolis-Hastings algorithm but is otherwise rejected.^49,50^ Similar moves are repeated until a satisfactory match is obtained between the model system and the experimental data. In this way a structure is generated that is consistent with experiment and should therefore be representative of the original material.

Here the RMC method is used, but rather than fitting the model system to an experimentally determined RDF, the pair-correlation functions, for each species pair, obtained from the MD quenches were used as the inputs to RMC, with the aim of generating much smaller cells suitable for DFT.

This method of combining MD derived structural data with RMC, to provide structures for DFT, has been used with success by the authors to study doped ZnO.^51^ In comparison to this previous work, several refinements to the method have been made here. First, RMC runs were initialized with cells containing randomised coordinates. For the stoichiometric case these contained 96 atoms (Zr_32_O_64_). Secondly, after every 500 accepted Monte-Carlo moves, an energy minimisation was performed on the RMC structure using the same potential model as for MD. This was then used as the input for the next round of RMC tests. In this way, the RMC algorithm was made to converge more quickly. Optimized structures were only accepted when the energy difference between consecutive energy minimised structures was less than 0.01 eV. Finally, the O–Zr–O and Zr–O–Zr bond angle distributions were included in RMC as additional constraints to the system.

For each composition, twenty distinct RMC cells were generated for use in DFT by initiating each run with a different random seed. The rmc++ code (version 1.4) was used for all RMC runs.^52^ During RMC single atom displacements of up to 2 Å were made during each Monte-Carlo loop.

### 2.3 Density functional theory

The DFT method was used to structurally minimise the unit cells obtained using the MD + RMC process. The Vienna Ab-initio Simulation Package (VASP)^53^ was used with the projector augmented wave (PAW)^54^ library supplied with the software. The GGA-PBE exchange correlation functional^55^ was used with the highest number of electrons treated as valence supplied for each species.

The cut-off energy was set at 450 eV for all calculations with a smearing width of 0.1 eV (using Gaussian smearing). A 4 × 4 × 4 *k*-point grid was set automatically using the Monkhorst–Pack scheme. The self-consistent field (SCF) stopping criterion was set to 1 × 10^−4^ eV and the geometry optimization stopping criterion was set at 1 × 10^−3^ eV. Atomic positions and supercell size and shape were all free to fully relax.

Stoichiometry was varied in the supercells initially by varying the Zr : O ratio in the empirical calculations. This was carried out to provide a hyper-stoichiometric DFT modelled system containing 32 Zr atoms and 66 O atoms (ZrO_2.0625_) while the hypo-stoichiometric DFT model contained 32 Zr atoms and 62 O atoms (ZrO_1.9375_). Oxygen gas was modelled as a dimer in a large box in order to accurately assess the bond strength enabling calculations investigating the drive to accommodate both hyper-stoichiometry and hypo-stoichiometry in the amorphous ZrO_2_ system.

Monoclinic ZrO_2_ was modelled to compare the energies of amorphous ZrO_2_ with the crystalline form – as well as understand the difference in energy and mechanism to accommodate deviations in stoichiometry compared to the amorphous ZrO_2_ system. Deviations in stoichiometry in crystalline ZrO_2_ were performed in a supercell containing 96 lattice sites (32 ZrO_2_ units). Deviations in stoichiometry were considered by accounting for vacancies on all symmetrically distinct sites as well as the incorporation of interstitial species onto ten symmetrically distinct sites.

## 3 Results

### 3.1 Structure

**Fig. 2:**
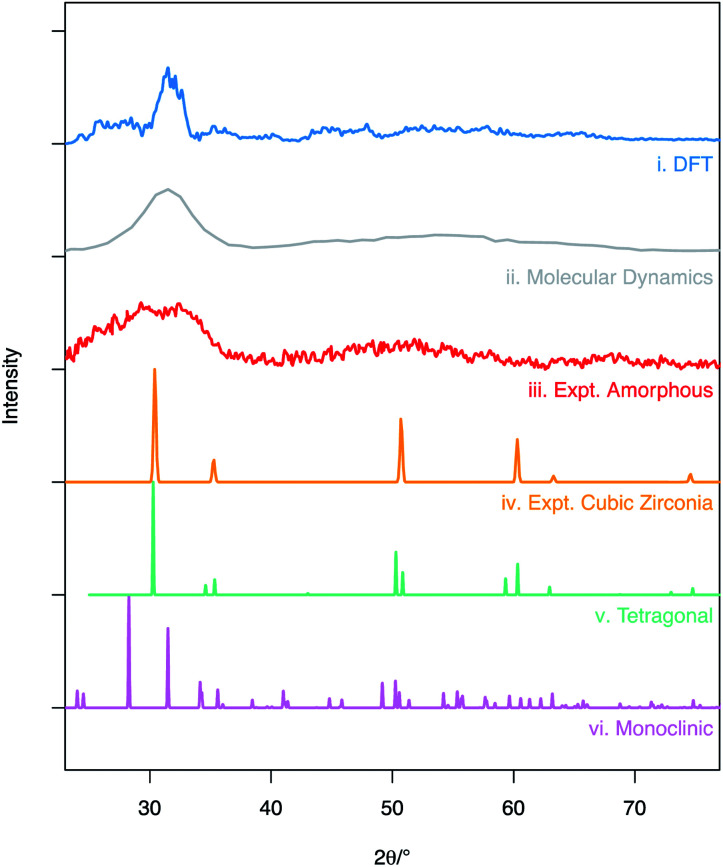
X-ray diffraction patterns obtained from simulated and experimental structures: (i) shows calculated XRD trace averaged over the 20 DFT structures, (ii) is the calculated XRD pattern for MD structure following melt-quench, (iii) shows the experimental XRD pattern for amorphous ZrO_2_ obtained by Sugiyama *et al.* at *T* = 423 K,^56^ (iv, v and vi) are the patterns for the cubic,^44^ tetragonal^45^ and monoclinic^43^ polymorphs of ZrO_2_ respectively (Cu K-α X-rays used across all datasets). The simulated diffraction patterns (i and ii) were obtained using the user/diffraction package in LAMMPS.^36,57^

A simulated XRD pattern was created for the amorphous ZrO_2_ structure obtained from the molecular dynamics melt-quench routine (Fig. 2(ii) – grey pattern). When compared to experimental data by Sugiyama *et al.* (Fig. 2(iii) – red pattern) the patterns can be seen to be similar – showing a broad, amorphous hump with at peak at ∼30° and a second, lower intensity hump for 2*θ* in the 40–60° range. This is a strong indication that the MD model is a good representation of the experimentally observed amorphous ZrO_2_ structure. The density of the amorphous structure generated by MD was 6.32 g cm^−3^.

The RMC derived supercells were relaxed using DFT. Simulated XRD patterns were then calculated for all twenty structures and then averaged to give the trace seen in Fig. 2(i). It can be seen that unlike the pattern generated from the supercell after the molecular dynamics routine, the features of the XRD patterns are sharper but still vary significantly from the fully crystalline ZrO_2_ polymorphs Fig. 2(iv–vi). It is expected that this difference in pattern profile can be attributed to a number of factors including: the limited size of the supercell causing imaging effects near 10 Å distances; the use of a relatively small total number of ZrO_2_ units (640 – limiting the ability to completely simulate a bulk amorphous solid); and the static nature of the simulations that results in a lack of thermal effects. The average density for the supercells after relaxation was 5.95 g cm^−3^.

**Fig. 3:**
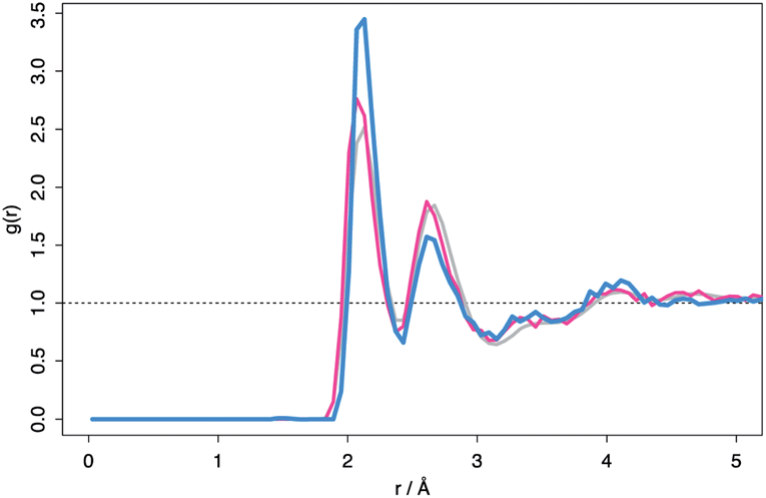
Radial distribution functions, *g*(*r*), of simulated amorphous zirconia (where *r* is atomic separation). Grey data: full amorphous structure obtained through molecular dynamics. Pink data: average data from 20 × 96 atom cells produced through RMC technique. Blue data: average RDF for the 15 lowest energy relaxed supercells obtained through DFT.

**Fig. 4:**
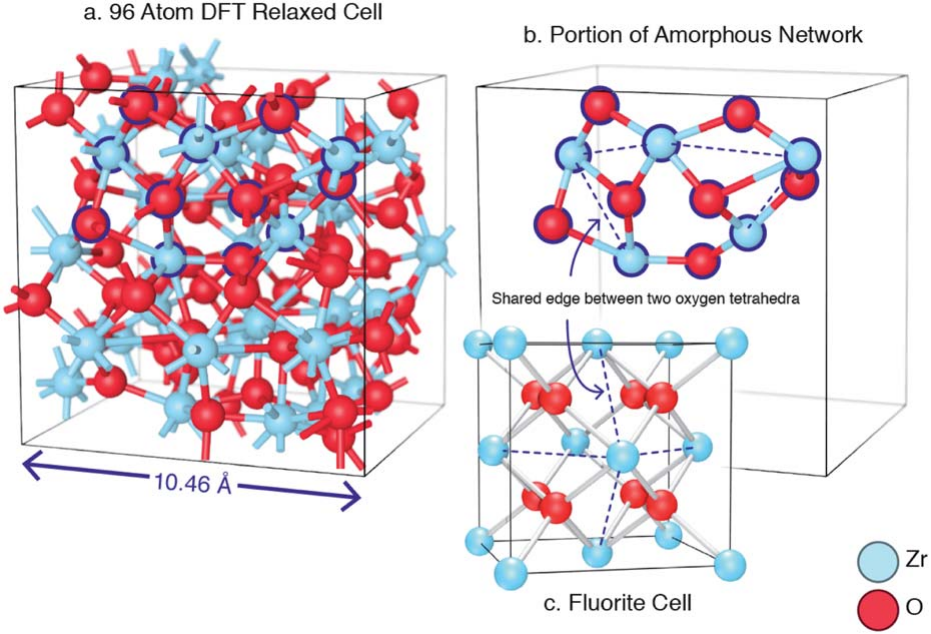
An example of a DFT relaxed amorphous ZrO_2_ cell. A section of the amorphous network corresponding to the outlined atoms from (a) is shown in sub-figure (b). This shows that the network is primarily composed of oxygen tetrahedra in edge sharing configurations. The dashed lines indicate the position of these shared edges. Sub-figure (c) shows that this edge sharing topology is also evident in the crystal polymorphs of zirconia.

Despite the differences in the patterns, the local structures in the DFT supercells compared to experimental are similar and therefore the local chemical effects can be reasonably predicted. The radial distribution function of the DFT data, the molecular dynamics data and analysis performed on experimental data are shown in Fig. 3 highlighting the similar local structures. Fig. 4 reports the similarity between the local morphology in the amorphous phase to the crystalline phase.

**Fig. 5:**
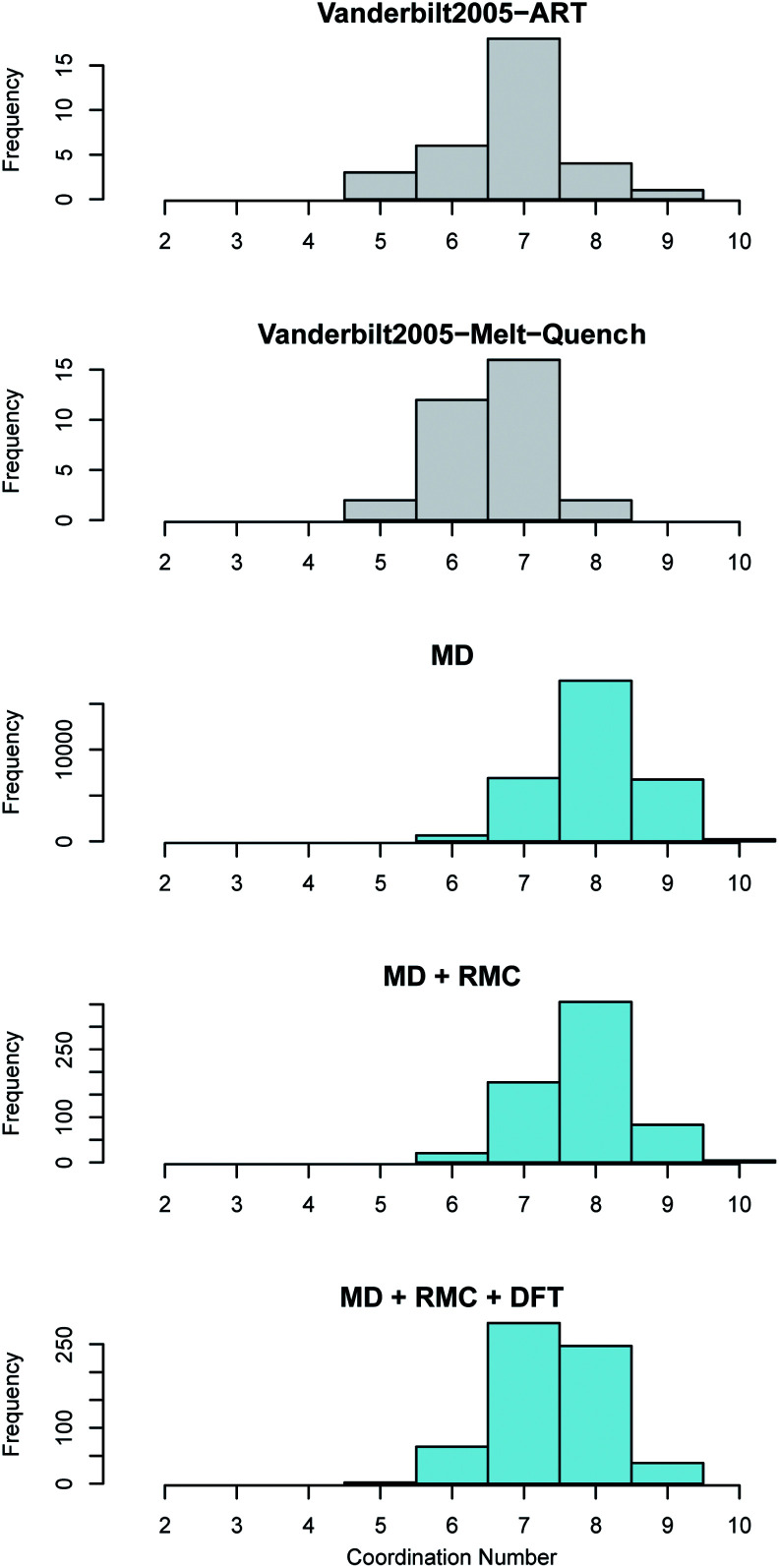
Zirconium coordination number distribution in amorphous ZrO_2_. The top two panes (shown in grey) reveal the distributions obtained by Vanderbilt *et al.*^34^ in previous work using the activation relaxation method and a 96 atom DFT melt-quench. The lower three panes (in blue) show those predicted by the current work. The MD results are for the 96 000 atom melt-quench with classical potentials. The MD + RMC distribution is averaged across the twenty RMC optimized cells (each containing 96 atoms). Finally, the MD + RMC + DFT results show the RMC cells following relaxation using DFT. Note, the considerable differences in the magnitude on the frequency axes is due to the varying total number of atoms considered for each method, not necessarily the supercell size (see Section 2).

In monoclinic ZrO_2_ the Zr is coordinated by 7 oxygen atoms whilst in both tetragonal and cubic ZrO_2_ the each Zr is coordinated by 8 oxygen atoms. Oxygen in the monoclinic polymorph is coordinated by either 3 or 4 Zr atoms whilst in the tetragonal and cubic polymorphs each O is coordinated by 4 Zr atoms. The coordination of both the Zr atoms in the amorphous ZrO_2_ structures generated in this work was compared to the previous work reported by Vanderbilt *et al.*^34^ in Fig. 5. Bonds were considered up to a cut-off distance of 3 Å to aid comparison with the previous work.

**Fig. 6:**
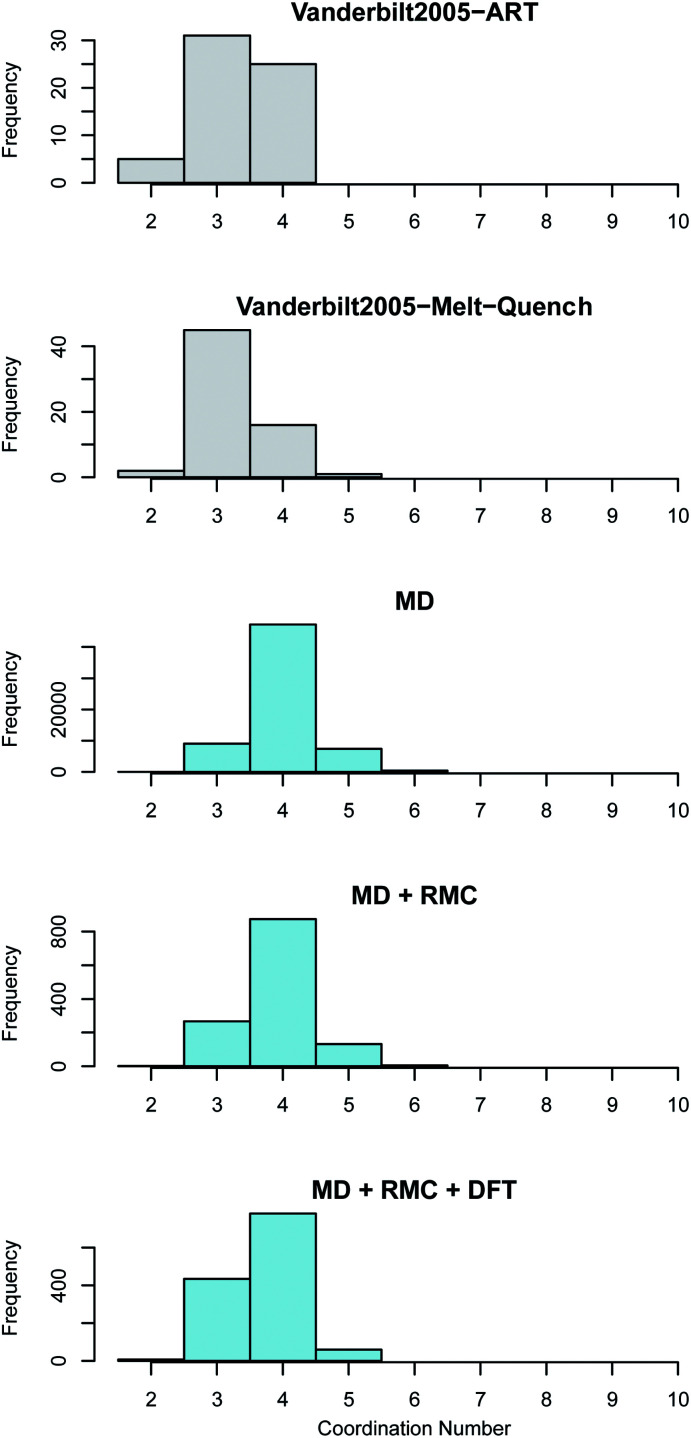
Oxygen coordination number distribution in amorphous ZrO_2._ The top two panes (shown in grey) reveal the distributions obtained by Vanderbilt *et al.*^34^ in previous work using the activation relaxation method and a 96 atom DFT melt-quench. The lower three panes (in blue) show those predicted by the current work. The MD results are for the 96 000 atom melt-quench with classical potentials. The MD + RMC distribution is averaged across the twenty RMC optimized cells (each containing 96 atoms). Finally, the MD + RMC + DFT results show the RMC cells following relaxation using DFT. Note, the considerable differences in the magnitude on the frequency axes is due to the varying total number of atoms considered for each method, not necessarily the supercells size (see Section 2).

The average coordination of both Zr and O in the current work is higher than the reported coordination values in the work of Vanderbilt *et al.* who considered the same number of atoms in each DFT supercell as were used in this work^34^ (see Fig. 5 and 6). The MD + RMC + DFT data from this work possesses a lower average coordination relative to the MD and MD + RMC generated amorphous ZrO_2_ structures in this work indicating that the smaller system size and/or the change in atomic description may reduce the average Zr coordination. Similarly, the oxygen coordination in the work of Vanderbilt *et al.*^34^ (a combination of 3 and 4) is more akin to the coordination environment in the monoclinic phase, whilst the oxygen coordination in the structures from this work are predominantly 4 coordinated, akin to the tetragonal and cubic polymorphs of ZrO_2_.

### 3.2 Deviations in stoichiometry

The ability for amorphous ZrO_2_ to accommodate deviations in stoichiometry is now investigated and compared to the crystalline ZrO_2_ systems. Deviations in stoichiometry in ionic materials impact a number of key properties including intrinsic diffusion mechanisms, mechanical properties and thermal transport properties. It is not expected that Zr will oxidize to a higher charge state than its formal 4+ charge in the stoichiometric ZrO_2_ system.

For excess oxygen, one may expect the formation of neutral oxygen defects such as the peroxide ion. The peroxide defect is an oxygen interstitial that combines with an oxygen on a lattice site to form an O_2_^2−^ ionic species where the two constituent oxygen atoms are covalently bonded at a distance of 1.49 Å from each other. The strong covalent bond is distinct from the other ionic bonding where it has been previously reported.^22^

Twenty hyper-stoichiometric (ZrO_2.0625_) amorphous supercells were relaxed using the same parameters as the stoichiometric supercells allowing the following simple reaction to be considered:

1 O_2_ + Zr_32_O_64_ → Zr_32_O_66_

**Fig. 7:**
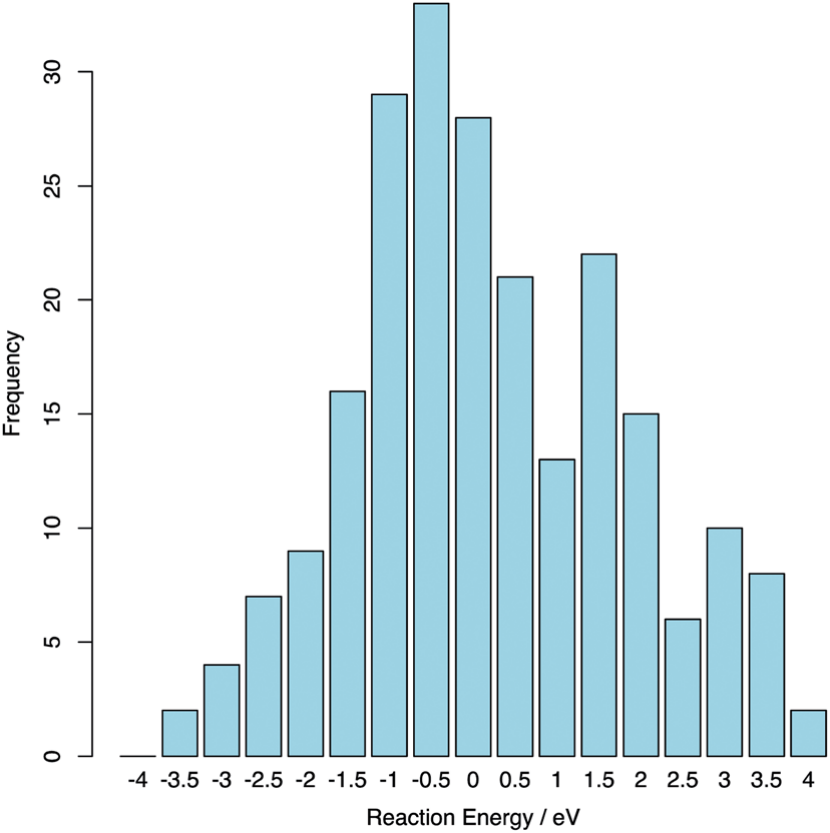
Histogram illustrating the reaction energy for O_2_ to enter the amorphous ZrO_2_ supercells.

The reaction was computed between each of the 15 lowest energy stoichiometric supercells and the 15 lowest energy hyper-stoichiometric supercells: totalling 225 reactions. The average reaction energy was 0.02 eV (0.01 eV per extra oxygen) indicating that excess oxygen can be readily accommodated into the structure *via* this interstitial-like topological feature. The range of energies is large (see Fig. 7) indicating that portions of amorphous ZrO_2_ will accommodate excess oxygen exothermically.

**Fig. 8:**
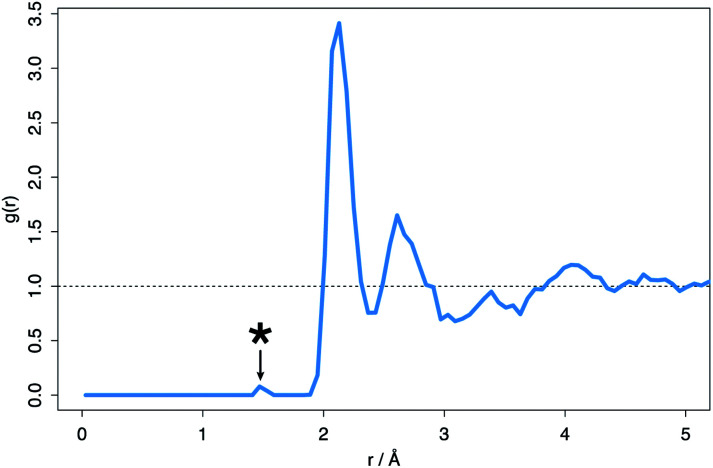
Average radial distribution function (RDF) for the hyper-stoichiometric ZrO_2_ system after DFT structural minimization. *Indicates the peak at 1.49 Å typical of the peroxide ion.

The fifteen most stable hyper-stoichiometric amorphous structures were all found to accommodate either one or both of the excess oxygen species through the formation of a peroxide ion, characterized by the 1.49 Å bond. This bond is identifiable on the average RDF pattern for the hyper-stoichiometric system (Fig. 8).

Accommodation of excess oxygen in crystalline, monoclinic ZrO_2_ was investigated to compare to the amorphous system. It was found that an excess oxygen atom preferentially sits at a (0.21, 0.44, 0.16) interstitial site which bonds to the (0.05, 0.314, 0.354) oxygen lattice site to produce a peroxide ion (as reported by Lyons *et al.*^58^). The enthalpy required to take ½O_2_ into solution was computed to be 1.09 eV – significantly larger than the value for solution into the amorphous structure.

Hypo-stoichiometry in amorphous ZrO_2_ was investigated in a similar manner to hyper-stoichiometry. Twenty supercells with 32 Zr atoms and 62 O atoms were created using the same routine using data from a molecular dynamics simulation. The following reaction was then considered to understand the drive to release oxygen:

2 Zr_32_O_64_ → Zr_32_O_64_ + O_2_

The average reaction energy for this (considering the 15 lowest energy structures) was 10.46 eV or 5.23 eV per oxygen removed. The energy to produce an oxygen vacancy in crystalline monoclinic ZrO_2_ was computed to be 11 eV, meaning that hypo-stoichiometry in amorphous ZrO_2_ is preferred, despite the significant energy penalty for hypo-stoichiometry in either system.

The predicted energy penalty to go from monoclinic ZrO_2_ to amorphous ZrO_2_ is 0.54 eV per ZrO_2_ unit. This energy is lower when considering deviations in stoichiometry: the energy penalty per ZrO_2.0625_ unit to go from crystalline to amorphous is 0.48 eV implying that the presence of any excess oxygen in crystalline ZrO_2_ will aid the formation of an amorphous phase when exposed to radiation damage. The same was found when considering hypo-stoichiometry – the energy penalty for a ZrO_1.9375_ unit to go from crystalline to amorphous was computed to be 0.45 eV. Larger deviations from stoichiometry are expected to reduce the energy penalty for amorphization, accordingly.

## 4 Summary

The amorphous structure of ZrO_2_ has been modelled using a combination of empirical potentials, reverse Monte-Carlo refinement and density functional theory. This produces results that compare well with previous atomic scale descriptions of the system. The coordination environment in the present work is more closely related to the tetragonal and cubic polymorphs of ZrO_2_, whilst the structures reported in the work of Vanderbilt *et al.*^34^ can be regarded as more similar to the coordination environment in the low temperature monoclinic polymorph. It should be noted that Vanderbilt *et al.* conducted their quenches exclusively using DFT which would have the advantage of being able to capture the low temperature monoclinic form. The use of DFT imposes some computational limitations, especially related to system size and the need for very rapid quench rates. By comparison our method allows large system sizes and considerably slower, more reasonable, quench rates (although still far higher than experiment). These methodological differences will lead to slight differences in the final structures but are unlikely to have a major consequence on the conclusions related to stoichiometry.

Unlike silica based glasses which consist of corner-sharing SiO_4_ tetrahedra, amorphous zirconia can be defined by tetrahedra in edge sharing configurations (akin to chalcogenide glasses^59^) as illustrated in Fig. 4. Previous investigations have focused on how the edge-sharing topology of chalcogenide glasses may change their behaviour in comparison to corner sharing glasses,^60,61^ these have included molecular dynamics studies.^62^ Amorphous TeO_2_ has been reported to be edge sharing by Brady *et al.*^63^ who also discuss the glass forming ability of corner-sharing glasses with edge-sharing and face-sharing glass and the increasing difficulty in glass formability with increasing structural restriction. The edge sharing nature of amorphous ZrO_2_ is consistent with the low amorphous to crystalline transition temperature observed experimentally.^9^

It is predicted that deviations in the stoichiometry are more readily accommodated in amorphous zirconia compared to the crystalline structure. The results suggest that the same mechanism accommodates hyper-stoichiometry in both the crystalline and amorphous systems but the morphological differences in the a-ZrO_2_ system have enabled the O_2_^2−^ dumbbell to be accommodated more readily in the non-crystalline form. It is expected that electronic disorder will also play a role in the accommodation of non-stoichiometry (especially with charged extrinsic defects)^25^ and may be a fruitful avenue for further work.

In a situation where an amorphous region of material is in contact with crystalline material, it is expected that the amorphous region will getter deviations in stoichiometry assuming the migration of the defects is kinetically viable. This is particularly relevant when considering amorphous phases that may exist at grain boundaries induced by radiation damage – for example in the semi-protective layer that forms as a result of oxidation of zirconium alloys used as cladding materials in light water reactors (LWRs). Migration of neutral peroxide species through grain boundaries may be the route for Zr metal oxidation as oxygen mobility through the bulk is slow and deviations from stoichiometry are very unfavourable. The presence and evolution of glassy phases along grain boundaries may therefore impact the oxidation behaviour of alloys with zirconia semi-protective layers. The same behaviour is expected for other HCP metals such as titanium alloys and warrants further work.

It is unclear whether a general rule can be formulated to understand whether or not an amorphous oxide system is able to deviate in stoichiometry. Other ionic glass network components such as Al, Ti, Be and Zn may be expected to behave similarly to amorphous ZrO_2_ compared with the more covalent Si and Ge glasses. The influence of multiple network formers, modifiers and intermediates is also likely to impact the overall propensity for a glassy system to vary its oxygen stoichiometry.

## Conflicts of interest

There are no conflicts to declare.

## Supplementary Material
